# Does Direct-to-Consumer Personal Genetic Testing Improve Gynecological Cancer Screening Uptake among Never-Screened Attendees? A Randomized Controlled Study

**DOI:** 10.3390/ijerph182312333

**Published:** 2021-11-24

**Authors:** Miki Watanabe, Satoyo Hosono, Hiroko Nakagawa-Senda, Sachiyo Yamamoto, Masami Aoyama, Satoru Hattori, Tamaki Yamada, Sadao Suzuki

**Affiliations:** 1 Department of Public Health, Nagoya City University Graduate School of Medical Sciences, Nagoya 467-8601, Japan; mwata911@med.nagoya-cu.ac.jp (M.W.); nakagawa@med.nagoya-cu.ac.jp (H.N.-S.); ssuzuki@med.nagoya-cu.ac.jp (S.S.); 2 Division of Cancer Screening Assessment and Management, Institute for Cancer Control, National Cancer Center, Tokyo 104-0045, Japan; 3 Health Promotion Division, Okazaki City Public Health Center, Okazaki 444-8545, Japan; yamamoto.sachiyo@city.okazaki.lg.jp (S.Y.); aoyama.masami@city.okazaki.lg.jp (M.A.); 4 Okazaki City Public Health Center, Okazaki 444-8545, Japan; hattori.satoru@city.okazaki.lg.jp; 5 Okazaki City Medical Association, Public Health Center, Okazaki 444-0875, Japan; t-yamada@okazaki-med.or.jp

**Keywords:** cancer screening, breast cancer, cervical cancer, screening uptake, genetic testing, randomized controlled trial

## Abstract

The clinical impact of direct-to-consumer genetic testing (DTC-GT) on health behavior change has remained controversial. The aim of this study is to clarify the short-term effects of DTC-GT on gynecological cancer screening uptake among middle-aged never-screened Japanese women in a randomized controlled trial (RCT). A total of 144 women aged 45–50 who had never undergone gynecological cancer screening were randomly selected to receive health education (control group), or health education and DTC-GT (intervention group), at a 1:1 ratio. We compared the gynecological screening uptake during the follow-up period. Furthermore, to estimate the impact of learning of an elevated genetic cancer risk in the intervention group, we conducted an analysis dichotomized by genetic risk category. A total of 139 women completed the one-year follow-up survey (69 in the control group and 70 in the intervention group). The follow-up period did not differ between control and intervention groups (the median follow-up period was 276 days and 279 days, respectively, *p* = 0.746). There were 7 (9.7%) women in the control group and 10 (13.9%) in the intervention group who attended breast cancer screening (*p* = 0.606), and 9 (12.5%) women from both groups attended cervical cancer screening (*p* = 1.000). Likewise, there were no significant differences in cancer screening uptake in the analysis stratified by risk category within the intervention group. In conclusion, there was no significant effect of DTC-GT on gynecological cancer screening uptake in this RCT setting. Increasing cancer screening attendance may require a combination of well-established intervention strategies and DTC-GT. Clinical Trial Registration: UMIN-CTR Identifier, UMIN000031709.

## 1. Introduction

Breast and cervical cancer are the leading and tenth-most common cancers among Japanese women, respectively [1]. In particular, the incidence of both cancers among young and middle-aged women is higher than that of other malignancies [1,2]. Nevertheless, the Organization for Economic Co-operation and Development (OECD) statistics show that breast and cervical cancer screening levels are as low as about 40% in Japan [3], despite the use of standard invitations and recall systems. In general, cancer screening programs must achieve high compliance to be effective and efficient. From a public health perspective, non- and under-attendees to cancer screening should be paid more attention because this population is more likely to have a low intention of screening, high levels of anxiety, and concurrent social and medical problems [4]. The Cabinet Office of the Government of Japan conducted a survey regarding cancer control in 2016, in which a great deal of under-screened women had economic, emotional, and cognitive barriers to cancer screening utilization [5]. Middle-aged women especially, are prone to have various barriers to participation in cancer screening. To motivate these individuals to undergo cancer screening, we focused on the receipt of personalized cancer risk predictions by direct-to-consumer genetic testing (DTC-GT).

Recent developments in genomic evidence and technology have led to the introduction of commercial genomic profiling services. DTC-GT, a service that consumers can purchase directly from private companies, provides consumers with genomic profiling for assessment of disease susceptibility. Several studies have evaluated the psychological, behavioral, and clinical impact of DTC-GT [6,7,8,9,10]. Gray et al. indicated that most study participants were interested in learning about their genetic risk for cancer [8]. Nielsen et al. demonstrated that receipt of DTC-GT information may be associated with modest positive lifestyle changes [10]. In contrast, other studies have indicated that receipt of genetic testing information does not significantly change diet, exercise, advanced care planning, or cancer screening behaviors [6,7,9,11]. The design of these studies was a cohort study. Therefore, the clinical utility of DTC-GT for health behavior change has remained controversial.

The aim of this randomized controlled trial (RCT) was to clarify the short-term effects of DTC-GT on breast and cervical cancer screening uptake among middle-aged never-screened Japanese women.

## 2. Materials and Methods

### 2.1. Study Design

This RCT was conducted in Okazaki City, Aichi Prefecture, Japan, and targeted middle-aged never-screened women for both breast and cervical cancer screening. The study was approved by Nagoya City Graduate School of Medical Sciences, Nagoya City University Hospital Institutional Review Board (approval no. 46-17-0015), and registered with the University Hospital Medical Information Network in Japan (UMIN000031709).

### 2.2. Study Participants

Using the database of a population-based primary cancer screening program (Resident Healthcare Information System) managed by Okazaki City Public Health Center on 6 February 2018, we identified 8188 women aged 45–49 years who had not undergone breast or cervical cancer screening in the past 10 years. A total of 6300 randomly selected women were invited by mail to participate in this study, of whom 414 responded. The researchers contacted these women via telephone or mail to check their study eligibility. The inclusion criteria were asymptomatic women aged 45–49 years and living in Okazaki City on 31 March 2018 who had never participated in breast or cervical cancer screening, and could connect to the internet without assistance. Women with any type of cancer and those who did not agree to participate in the follow-up survey were excluded.

**Figure 1:**
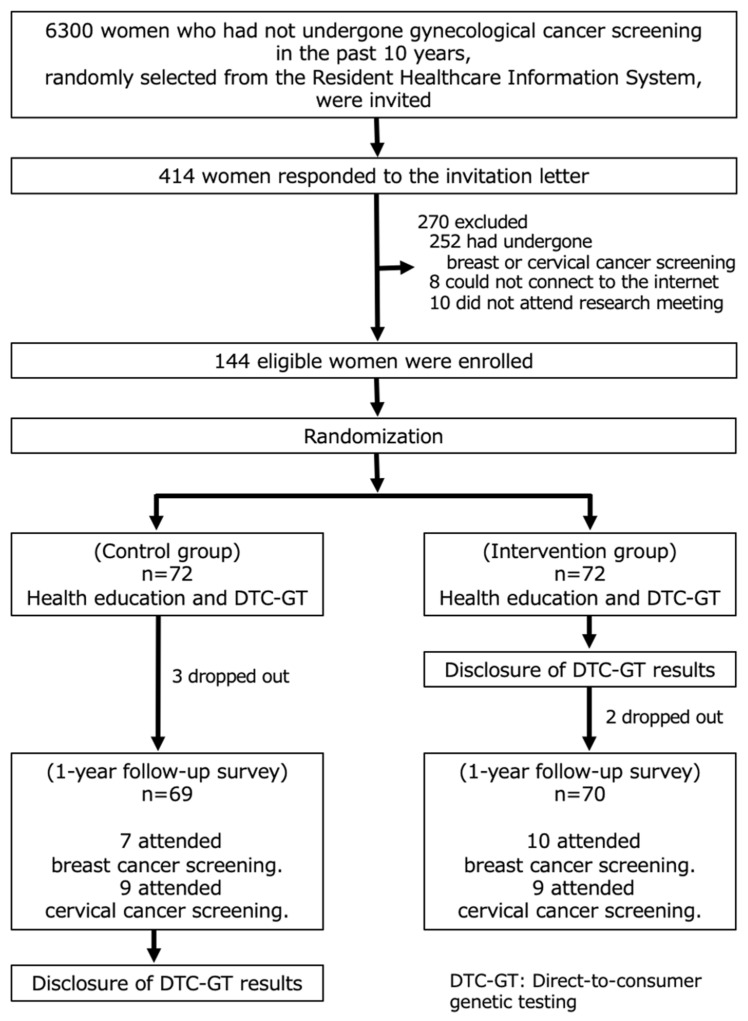
Study flowchart, in Okazaki, Japan, 2018–2019.

Of 414 respondents, 154 women met the inclusion criteria; 252 had undergone breast or cervical cancer screening, or comprehensive checkup, at their worksite, and 8 could not connect to the internet. Ten women who did not attend the research meeting were excluded, leaving 144 subjects who participated in this study (Figure 1). Okazaki City Public Health Center sent an invitation for cancer screening in May 2018 and a reminder letter in November 2018.

### 2.3. Randomization

From March to June 2018, a research meeting was held for the study participants at the Okazaki City Medical Association, Harusaki Health Examination Center, to explain the study design and obtain consent for participation in the study. All women gave written informed consent. The 144 participating women were randomly allocated to either the control or intervention group at a 1:1 ratio using the sealed envelope method. Researchers and participants were aware of the group allocations. Regardless of group allocation, all study participants received free DTC-GT.

### 2.4. Data Collection

In the research meeting, all study participants completed a baseline questionnaire on demographic characteristics, including education, physical activity, sleep, alcohol consumption, smoking, dietary patterns, cancer history of parents and friends, risk perception for breast and cervical cancer, health literacy, self-rated health, happiness, intention to participate in cancer screening, attitude towards genetic testing, and reasons for never-attendance at gynecological cancer screening programs.

From February to May 2019, a one-year follow-up survey was implemented via mail, using a self-administered questionnaire. We collected the same information as that in the baseline survey, in addition to breast and cervical cancer screening attendance from the period between the baseline survey and completion of the one-year follow-up survey, which was defined as the follow-up period. In addition, we asked the attendees to provide reasons for participating in cancer screening.

### 2.5. Genetic Testing

For personalized cancer risk prediction, we used ‘MYCODE’ (DeNA Life Science, Inc., Tokyo, Japan), an online DTC personal genome service for the Japanese general population. MYCODE can provide information on an individual’s susceptibility to 38 types of sporadic cancers, including breast and cervical cancer [12].

Saliva samples from all participants were collected for genetic testing. Genotyping was performed using either Infinium OmniExpress-24+ BeadChip or Human OmniExpress-24+ BeadChip (Illumina Inc., San Diego, CA, USA) [13]. Cancer susceptibility was assessed using odds ratios (ORs) calculated by DeNA Life Science, Inc. [14]. In June 2019, DeNA Life Science, Inc. provided the researchers with the point estimate ORs for rs2981578 in *FGFR*2 [15] and rs4784227 in *LOC643714* [16] for breast cancer, and rs8067378 and rs9277952 [17] for cervical cancer for all study participants [18]. Based on the ORs of each polymorphism and their combinations, genetic risk groups were dichotomized for each cancer; ORs of the low-risk group were less than 1.0, and those of the high-risk group were greater than or equal to 1.0. ORs of 1.0 indicated the average risk among the general Japanese population (Figure S1).

### 2.6. Intervention

We provided all enrolled women with a short lecture on primary and secondary cancer prevention for breast and cervical cancer in the research meeting. MYCODE provided personalized cancer risk predictions according to genotype, and advice about cancer prevention on a webpage with access restricted to each user. The advice included recommended lifestyle habits for primary cancer prevention, cancer screening, and treatment, which was not personalized according to genotype. Participants in the intervention group were able to access and read the information at any time. In contrast, although genetic testing was also performed for the control group, their cancer risk prediction results were not uploaded for access online until completion of the one-year follow-up survey. Their results were made available online in May 2019. All participants, including the control group, gave written informed consent with the understanding that the disclosure of results for the control group would be one year later.

### 2.7. Statistical Analysis

The primary outcome was the difference in participation rate in breast and cervical cancer screening between the control and intervention groups during the follow-up period. For a sample size calculation, we assumed a 30% and 10% participation rate in the intervention and control groups, respectively, based on previous studies by Bloss et al. [6] and Fujiwara et al. [19]. Using the power of 80% and a two-sided alpha error level of 0.05, we calculated that 72 women were needed in each group.

The primary analysis was performed on an intention-to-treat basis. Age, in the baseline survey and follow-up period in the control and intervention groups, was compared using the Mann–Whitney U test. Differences in dichotomized variables between the two groups were assessed using Fisher’s exact test, including participation in cancer screening. To estimate the impact of learning of an elevated genetic cancer risk in the intervention group, we conducted an analysis dichotomized by genetic risk category. A *p*-value less than 0.05 was considered statistically significant.

All analyses were performed using the STATA version 13.0 (STATA Corporation, College Station, College Station, TX, USA).

### 2.8. Patient and Public Involvement

There was no patient or public involvement in the conception, design, or conduct of the study, or the writing and editing of this study.

## 3. Results

Of the 144 participants who completed the baseline survey, 69 (95.8%) women in the control group and 70 (97.2%) in the intervention group completed the one-year follow-up survey (*p* = 1.000). The follow-up period ranged from 240 to 366 days (median, 276 days) in the control group, and from 229 to 342 days (median, 279 days) in the intervention group. There were no significant differences in the follow-up period (*p* = 0.746) (Table 1).

Baseline characteristics of the study participants are shown in Table 1. The median age in the control group was 47 years and that in the intervention group was 48 years. Approximately 30% of participants in both groups had high intention to participate in breast and cervical cancer screening in the baseline survey. There were no differences between the control group and intervention group in body mass index, length of education, cancer history of parents, cancer history of friends, or intention to participate in breast and cervical screening in the baseline survey.

The distribution of point estimates and ORs for each polymorphism locus and combined risk according to genetic testing results, are shown in Table 2 and Table S1. The number of women at high risk for breast cancer in the control and intervention groups was 34 (47.2%) and 45 (62.5%), respectively. The number of women at high risk for cervical cancer in the control and intervention groups was 42 (58.3%) and 35 (48.6%), respectively. There was no significant difference in the proportion of women in each genetic risk category for breast or cervical cancer between the control and intervention groups.

The participation rate in breast and cervical cancer screening in the one-year follow-up survey is shown in Table 3. Of the 70 participants in the intervention group who completed the one-year follow-up survey, 62 (88.6%) assessed their personalized cancer risk prediction on the webpage. For breast cancer screening, seven (10.1%) women in the control group and 10 (14.3%) in the intervention group attended screening (*p* = 0.606). In the intervention group, two (8.0%) women in the low-risk group and eight (17.8%) in the high-risk group attended breast cancer screening (*p* = 0.314). For cervical cancer screening, nine (13.0% and 12.9%, respectively) women in both groups attended screening (*p* = 1.000). In the intervention group, five (13.9%) women in the low-risk group and four (11.8%) women in the high-risk group (*p* = 1.000) attended cervical cancer screening. There were no significant differences in cancer screening uptake between the control and intervention groups. Likewise, there were no significant differences in cancer screening uptake in the analysis stratified by risk category within the intervention group.

## 4. Discussion

This RCT investigated the impact of DTC-GT on gynecological cancer screening uptake among never-screened Japanese women. There was no significant difference in cancer screening uptake between control and intervention groups. Similarly, there was no difference in cancer screening uptake in the analysis stratified by risk category based on DTC-GT.

Cancer screening is a key strategy for reducing cancer mortality, with marked reductions in mortality requiring high levels of screening uptake. To promote uptake, strategies should involve multiple factors, such as different target cancers, invitees, health-service settings, and screening tests [20]. In Japan, biennial mammogram screening for women aged 40 or above and pap smear testing for women aged 20 or above are used to detect gynecological cancers at early stages [21,22]. However, breast and cervical screening participation levels have remained low among Japanese women [3]. Lu et al. suggested various barriers to participation in breast and cervical cancer screening, including cognitive, emotional, economic, logistical, and social barriers [23]. Given the cultural backgrounds of Asian women, gynecological cancer screening may present a heavy emotional burden in the form of embarrassment or anxiety, compared with other types of cancer screening. In our study, the most prevalent reasons for never-attendance were emotional barriers, such as pain and anxiety (63.2% at baseline). On the other hand, the prevalence of cognitive barriers among our study participants was relatively low, with 73.6% of participants indicating that they understood the necessity for cancer screening, even among those currently in good physical condition (Figure S2). Personalized disease risk prediction by DTC-GT is expected to affect cognitive but not emotional barriers. Therefore, DTC-GT could not improve breast or cervical cancer screening attendance in this RCT setting.

Bloss et al. reported that DTC-GT did not result in any short-term changes in psychological health, diet, exercise behavior, or participation in screening tests. They speculated that these findings were attributed to the failure of 44% of subjects to complete the follow-up survey, or the subjects’ knowledge of DTC-GT. In addition, they conducted a longitudinal cohort study of selected subjects from health and technology companies, who may not be representative of the general population [6]. Gray et al. also assessed participants’ behavior after DTC-GT using a longitudinal study design. Of 456 female participants, 12% received an elevated breast cancer genetic risk score. The participation rate in breast cancer screening among the elevated risk group during the six months from enrollment was 19.2%, versus that in the average or reduced risk group of 27.2%. They concluded that elevated cancer risk estimates by DTC-GT did not significantly change cancer screening behaviors. In the study by Gray et al., participants older than 50 years accounted for 36% of the sample, and were more likely to participate in cancer screening at baseline [9]. Our RCT study differed from several preceding studies in study design, sample size, completion of follow-up, and the characteristics of participants, including never-screened and middle-aged women. In addition, the control group completed the same test as the intervention group, but simply did not receive their test results until completion of the one-year follow-up survey. Therefore, DTC-GT may be an insufficient incentive for the intervention group.

Another factor that may have contributed to our negative findings is participants’ comprehension of the DTC-GT results. Our study participants were provided their point estimate ORs, which ranged from 0.69 to 1.85 for breast cancer and from 0.75 to 1.43 for cervical cancer (Table S1). DTC genetic risk prediction performance, which is based on a combination of variants derived from genome-wide association studies and the heritability of each risk variant included in DTC-GT, was limited. Therefore, it was difficult for our participants to accurately interpret the DTC-GT results without assistance, which might in turn have contributed to the low screening attendance (Figure S1).

In general, an individual’s response to a call for cancer screening is influenced by personal factors, including the perceived risk of cancer, self-efficacy, and intention to participate. According to the theory of planned behavior, one of the strongest determinants of behavior is a person’s intention to perform that behavior [24]. In fact, among our study subjects, participation by those with high intention to undergo cancer screening attended breast and cervical cancer screenings consistently more often than those with low intention, independently of whether or not they received the intervention of a DTC genetic risk prediction (Table S2). Therefore, both an intervention of DTC-GT and high intention to participate in screening at baseline may be required to indicate screening uptake behavior. These findings revealed the importance of encouraging individuals to participate in screening in advance of DTC-GT. A combination of DTC-GT and other strategies for screening uptake, including mailed interventions, media campaigns, or health education [25], may be more likely to improve uptake than single strategies.

This study is the first RCT to investigate the clinical utility of DTC-GT among women who have never undergone gynecological cancer screening. Several limitations warrant mention. Firstly, the number of study participants was small and the dropout rate from follow-up was 3.5%. In fact, statistical power was not sufficient to determine the effect of DTC-GT. However, we were able to deduce that participation in screening did not differ according to whether or not participants received genetic risk information. Secondly, this study used self-reported screening uptake to measure outcomes. Besides recall bias, social desirability bias may have led to the over-reporting of screening uptake. However, these biases are expected to have affected both groups, suggesting that these effects were comparable between the two groups. Finally, the study participants might not be representative of general never-screened people due to the very low response rate. In fact, they had relatively high intention to participate in screening in the baseline survey. Their characteristics might lead to the overestimation of the effects of DTC-GT. The screening attendance as an intrinsic effect of DTC-GT may be lower than that observed in this study.

## 5. Conclusions

There were no significant differences in gynecological cancer screening uptake between those who did and did not receive DTC-GT predictions in this RCT. Without high intention to participate in screening in advance, DTC-GT may be insufficient to promote attendance. Further investigation of these findings is warranted in larger populations, possibly with DTC-GT and other intervention strategies for screening uptake.

## Figures and Tables

**Table 1:** Characteristics of study participants in Okazaki, Japan, 2018.

		Control Group (*n* = 72)		Intervention Group (*n* = 72)	
		*n*	(%)	*n*	(%)
Age at baseline (years)	45	13	(18.1)	7	(9.7)
	46	18	(25.0)	13	(18.1)
	47	13	(18.1)	10	(13.9)
	48	16	(22.2)	18	(25.0)
	49	9	(12.5)	21	(29.2)
	50	3	(4.2)	3	(4.2)
	Median	47		48	
	(Interquartile range)	(46–48)		(46–49)	
Body mass index (kg/m^2^)	<25	51	(70.8)	54	(75.0)
	≥25	21	(29.2)	18	(25.0)
Length of education	≤12 years or less	37	(51.4)	34	(47.2)
	>12 years	35	(48.6)	38	(52.8)
Cancer history of parents	Yes	30	(41.7)	25	(34.7)
	No	42	(58.3)	47	(65.3)
Cancer history of friends	Yes	24	(33.3)	27	(37.5)
	No	48	(66.7)	45	(62.5)
Intention to participate in cancer screening					
Breast cancer ^1^	Low intention	48	(66.7)	50	(69.4)
	High intention	24	(33.3)	22	(30.6)
Cervical cancer ^1^	Low intention	49	(68.1)	50	(69.4)
	High intention	23	(31.9)	22	(30.6)
Follow-up period (days) ^2^	Median (min, max)	276	(240, 366)	279	(229, 342)

^1^ Those with low intention included women who intended to participate in a cancer screening in a few years or did not intend to do so in the future. Those with high intention included women who intended to participate in a cancer screening within a few months or a year. ^2^ *p*-value was 0.746 by the Mann–Whitney U test.

**Table 2:** Distribution of participants according to genetic risk group for breast and cervical cancer in Okazaki, Japan, 2018.

		Control Group (*n* = 72)		Intervention Group (*n* = 72)		*Fisher*
Locus of Polymorphism	Risk Group ^1^	*n*	(%)	*n*	(%)	*p*-Value
**Breast Cancer**						
rs2981578	Low	51	(70.8)	51	(70.8)	
	High	21	(29.2)	21	(29.2)	1.000
rs4784227	Low	46	(63.9)	38	(52.8)	
	High	26	(36.1)	34	(47.2)	0.237
Combination of rs2981578 and rs4784227	Low	38	(52.8)	27	(37.5)	
	High	34	(47.2)	45	(62.5)	0.094
**Cervical Cancer**						
rs8067378	Low	38	(52.8)	37	(51.4)	
	High	34	(47.2)	35	(48.6)	1.000
rs9277952	Low	50	(69.4)	55	(76.4)	
	High	22	(30.6)	17	(23.6)	0.453
Combination of rs8067378 and rs9277952	Low	30	(41.7)	37	(51.4)	
	High	42	(58.3)	35	(48.6)	0.316

^1^ Risk categories were determined as follows: low-risk group, odds ratio less than 1.0; high-risk group, odds ratio greater than or equal to 1.0. Odds ratio of 1.0 indicates the average risk among the general Japanese population.

**Table 3:** Participation in gynecological cancer screening during the follow-up period, stratified by intervention and total genetic risk.

		Control Group (*n* = 69) ^1^		Intervention Group (*n* = 70) ^1^							
				Overall		Low-Risk ^2^		High-Risk ^2^		*Fisher* ^3^	*Fisher* ^4^
Cancer Screening		*n*	(%)	*n*	(%)	*n*	(%)	*n*	(%)	*p*-Value	*p*-Value
Breast	Yes	7	(10.1)	10	(14.3)	2	(8.0)	8	(17.8)	0.606	0.314
	No	62	(89.9)	60	(85.7)	23	(92.0)	37	(82.2)		
Cervical	Yes	9	(13.0)	9	(12.9)	5	(13.9)	4	(11.8)	1.000	1.000
	No	60	(87.0)	61	(87.1)	31	(86.1)	30	(88.2)		

^1^ Three participants in the control group and two participants in the intervention group dropped out during the follow-up period. ^2^ Risk categories were determined as follows: low-risk group, odds ratio less than 1.0; high-risk group, odds ratio greater than or equal to 1.0. Odds ratio of 1.0 indicates the average risk among the general Japanese population. ^3^ Comparison between control and intervention groups. ^4^ Comparison between low-risk and high-risk groups within the intervention group.

## Data Availability

The datasets obtained and/or analyzed during the current study are available from the corresponding author upon reasonable request. The data are not publicly available due to participants’ privacy and consent.

## Supplementary Materials

The following are available online at https://www.mdpi.com/article/10.3390/ijerph182312333/s1, Figure S1: Sample of genetic risk for the participants, Figure S2: Reasons for never-attendance at gynecological cancer screening in the baseline survey in Okazaki, Japan, 2018, Table S1: Distribution of participants according to genetic risk for breast and cervical cancer, Table S2: Participation in gynecological cancer screening during the follow-up period, stratified by intervention and intention to participate in screening in the baseline survey.
