# Supplementary material for: Does Direct-to-Consumer Personal Genetic Testing Improve Gynecological Cancer Screening Uptake among Never-Screened Attendees? A Randomized Controlled Study
Source: Int J Environ Res Public Health. 2021 Nov 24;18(23):12333. doi: 10.3390/ijerph182312333 (PMC8657107; doi:10.3390/ijerph182312333)
Supplement: Supplementary file 1 [file ijerph-18-12333-s001.zip › Supplementary Files/ijerph_supfig1_20211116.pdf]

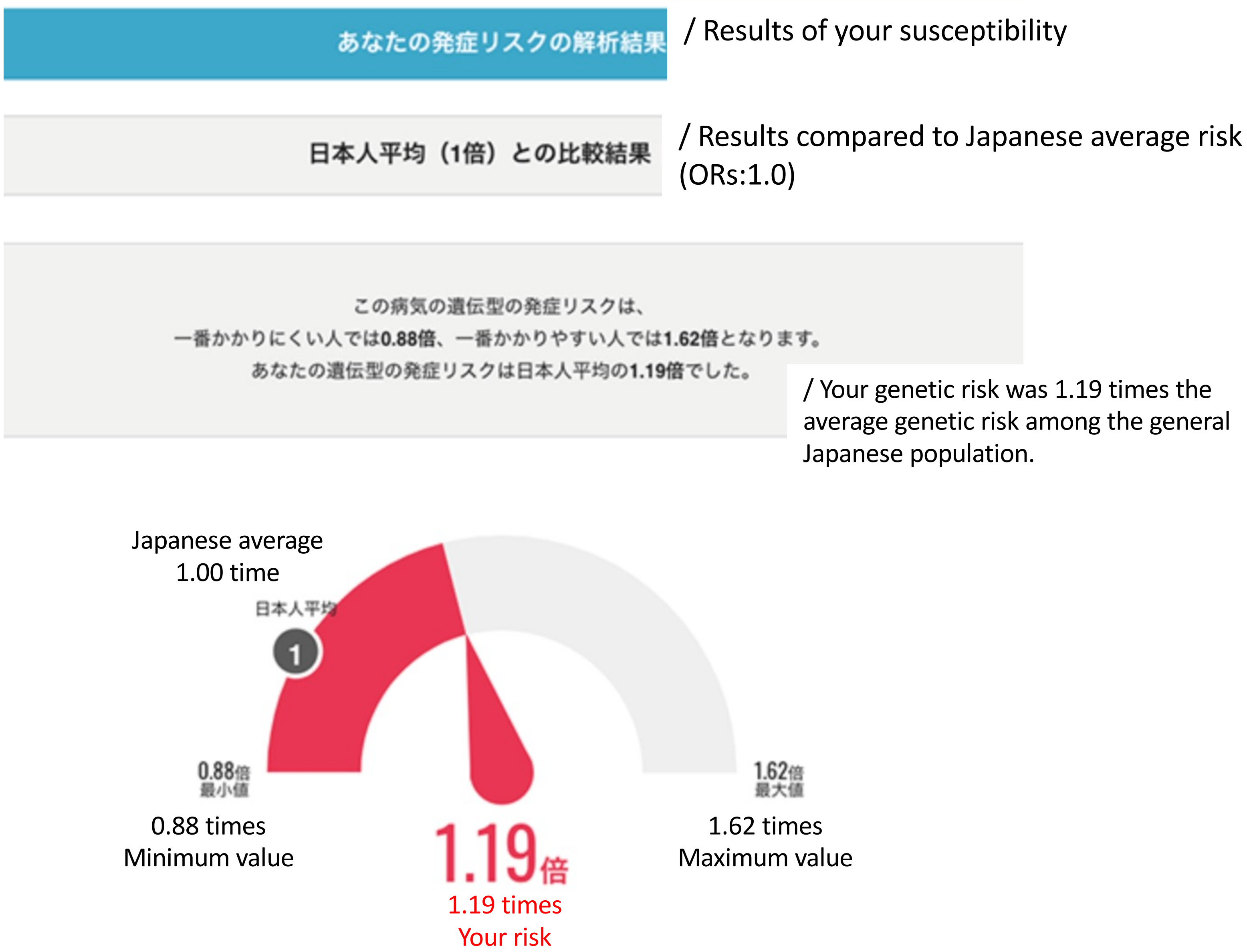

Figure S1. Sample of genetic risk for the participants

MYCODE :Sample-report https://mycode.jp/plans/sample-report.html
