# Supplementary material for: Does Direct-to-Consumer Personal Genetic Testing Improve Gynecological Cancer Screening Uptake among Never-Screened Attendees? A Randomized Controlled Study
Source: Int J Environ Res Public Health. 2021 Nov 24;18(23):12333. doi: 10.3390/ijerph182312333 (PMC8657107; doi:10.3390/ijerph182312333)
Supplement: Supplementary file 1 [file ijerph-18-12333-s001.zip › Supplementary Files/ijerph_supp tab1_20211013.pdf]

Table S1. Distribution of participants according to genetic risk for breast and cervical cancer

Locus of polymorphism	Odds ratio [1]	Control group (n=72)		Intervention group (n=72)		*Fisher*
		n	(%)	n	(%)	*p-value*
**Breast cancer risk**						
rs2981578	0.80	13	(18.1)	12	(16.7)	
	0.98	38	(52.8)	39	(54.2)	
	1.21	21	(29.2)	21	(29.2)	1.000
rs4784227	0.85	46	(63.9)	38	(52.8)	
	1.14	24	(33.3)	32	(44.4)	
	1.53	2	(2.8)	2	(2.8)	0.357
Combination of rs2981578 and rs4784227	0.69	10	(13.9)	9	(12.5)	
	0.84	25	(34.7)	15	(20.8)	
	0.92	3	(4.2)	3	(4.2)	
	1.03	11	(15.3)	14	(19.4)	
	1.13	13	(18.1)	22	(30.6)	
	1.38	8	(11.1)	7	(9.7)	
	1.51	0	(0)	2	(2.8)	
	1.85	2	(2.8)	0	(0)	0.250
**Cervical cancer risk**						
rs8067378	0.91	38	(52.8)	37	(51.4)	
	1.07	31	(43.1)	28	(38.9)	
	1.26	3	(4.2)	7	(9.7)	0.430
rs9277952	0.82	10	(13.9)	15	(20.8)	
	0.97	40	(55.6)	40	(55.6)	
	1.14	22	(30.6)	17	(23.6)	0.469
Combination of rs8067378 and rs9277952	0.75	5	(6.9)	6	(8.3)	
	0.88	25	(34.7)	31	(43.1)	
	1.03	30	(41.7)	20	(27.8)	
	1.04	1	(1.4)	1	(1.4)	
	1.22	10	(13.9)	13	(18.1)	
	1.43	1	(1.4)	1	(1.4)	0.648

[1] Odds ratio of 1.0 indicates the average risk among the general Japanese population.
