# Supplementary material for: Does Direct-to-Consumer Personal Genetic Testing Improve Gynecological Cancer Screening Uptake among Never-Screened Attendees? A Randomized Controlled Study
Source: Int J Environ Res Public Health. 2021 Nov 24;18(23):12333. doi: 10.3390/ijerph182312333 (PMC8657107; doi:10.3390/ijerph182312333)
Supplement: Supplementary file 1 [file ijerph-18-12333-s001.zip › Supplementary Files/ijerph_supp tab2_20211013.pdf]

Table S2. Participation in gynecological cancer screening during the follow-up period, stratified by intervention and intention to participate in screening in the baseline survey

		Overall (n=144)					Low intention [1]					High intention [2]				
		Low intention [1]		High intention [2]		*Fisher*	Control group		Intervention group		*Fisher*	Control group		Intervention group		*Fisher*
**Cancer Screening**		**n**	**(%)**	**n**	**(%)**	***p-value***	**n**	**(%)**	**n**	**(%)**	***p-value***	**n**	**(%)**	**n**	**(%)**	***p-value***
Breast	Yes	6	(6.3)	11	(25.0)		2	(4.3)	4	(8.3)		5	(22.7)	6	(27.3)	
	No	89	(93.7)	33	(75.0)	0.004	45	(95.7)	44	(91.7)	0.677	17	(77.3)	16	(72.7)	1.000
Cervical	Yes	9	(9.4)	9	(20.9)		6	(12.5)	3	(6.3)		3	(14.3)	6	(27.3)	
	No	87	(90.6)	34	(79.1)	0.098	42	(87.5)	45	(93.8)	0.486	18	(85.7)	16	(72.7)	0.457

[1] Those with low intention included women who intended to participate in a cancer screening in a few years or did not intend to do so in the future.

[2] Those with high intention included women who intended to participate in a cancer screening within a few months or a year.
